# Newcastle disease vaccine adoption by smallholder households in Tanzania: Identifying determinants and barriers

**DOI:** 10.1371/journal.pone.0206058

**Published:** 2018-10-24

**Authors:** Zoë A. Campbell, Thomas L. Marsh, Emmanuel A. Mpolya, Samuel M. Thumbi, Guy H. Palmer

**Affiliations:** 1 Paul G. Allen School for Global Animal Health, Washington State University, Pullman, Washington, United States of America; 2 Nelson Mandela African Institution of Science and Technology (NM-AIST), Arusha, Tanzania; 3 School of Economic Sciences, Washington State University, Pullman, Washington, United States of America; 4 Kenya Medical Research Institute (KEMRI), Kisian, Kenya; The University of Melbourne, AUSTRALIA

## Abstract

**Background:**

Food security is critical to achieving sustainable growth, poverty reduction, and political and economic stability. Livestock have the potential to improve the food security of smallholder households in developing countries, but livestock productivity is constrained by disease. The extent to which households adopt innovations such as vaccines impacts disease control; however, the behavioral and economic drivers underlying household decisions to adopt or forgo vaccination are not well understood. We address this gap with a study of adoption of Newcastle disease (ND) vaccines by chicken-owning households in Tanzania.

**Methods:**

A cross-sectional survey was administered to 535 households owning indigenous chickens in Arusha, Singida, and Mbeya regions in Tanzania. We measured potential predictors of ND vaccine adoption including knowledge, attitudes, and practices. Logistic regression was used to identify predictors correlated with three stages of household adoption: awareness of ND vaccines, previous vaccination, and recent vaccination (within four months) consistent with veterinary guidelines.

**Results:**

Eighty percent of households were aware of ND vaccines, 57% had previously vaccinated, and 26% had recently vaccinated. Knowing someone who vaccinated increased the odds of a household previously vaccinating [adjusted odds ratio (AOR): 1.32, 95% CI: 1.1–1.5]. Larger flock size was also associated with higher odds of previous vaccination (AOR: 1.03 for a one chicken increase, 95% CI: 1.01–1.05). Usage of traditional medicine decreased the odds of previously vaccination (AOR: 0.58, 95% CI: 0.36–0.95).

**Conclusion:**

Our findings suggest that encouraging the flow of professional-level knowledge within the community by vaccine adopters is a strategy to increase vaccine adoption. Enhancing local chicken productivity through increased vaccine coverage would strengthen a key smallholder household resource for food and economic security.

## Introduction

Food security is critical to achieving sustainable growth, poverty reduction, and political and economic stability, critically so in regions undergoing rapid population growth [1]. Smallholder households, characterized by having access to fewer resources relative to other farmers in the sector, have a crucial role in the food security of developing countries [2]. Smallholder households provide up to 80% of the food supply in Asia and sub-Saharan Africa through farming and livestock production [3]. Livestock generate income in smallholder households and serve as source of protein and micronutrients [4]. The potential for livestock to contribute to improving food security, increasing incomes of poor households, and reducing poverty is significant, but these benefits cannot be realized without the control of disease.

The extent to which livestock-owning households make use of veterinary best practices and technology such as vaccines has a huge impact on the control and prevention of disease, but the behavioral and economic drivers underlying household decisions to adopt or forgo vaccination are not well understood. We address this gap with a study of adoption of Newcastle disease (ND) vaccines by chicken-owning households in Tanzania.

Chickens are one of the most common livestock species owned because they provide food, generate income, improve social status, and are a highly liquid household asset [5]. Chickens are less expensive to acquire and maintain than other livestock, and women more commonly make management and investment decisions about chickens compared to other livestock [6,7]. In low-income, food-deficit countries, production of local chickens raised by rural households is estimated to represent 70% of total chicken production [5]. Efficient production is constrained by Newcastle disease, an infectious and virulent disease that causes high mortality in poultry [8]. Newcastle disease is endemic in areas of Central and South America, and widely spread in Asia, the Middle East, and Africa [9]. Newcastle disease has no treatment, but it can be effectively controlled through vaccination [9–11]. Vaccination decreases chicken mortality and results in increased flock size in smallholder production systems, but despite this, the vaccination rate outside of the commercial sector remains low [10,12]. This low vaccine uptake persists despite the development of the thermotolerant I-2 vaccine designed to overcome the need for a cold chain [10]. Our study identifies factors underlying household decisions to adopt or forgo ND vaccination, addressing a significant gap in knowledge relevant to improving vaccine coverage in livestock in smallholder settings.

## Materials and methods

In this section, we summarize the theory, survey design, survey administration, and data analyses used in this study. A cross-sectional survey designed to identify determinants and barriers of ND vaccine uptake was administered to 535 households across Arusha, Singida, and Mbeya regions in Tanzania. The sampling framework was designed to capture the variability in socioeconomic status, the structure of veterinary services, and vaccine access of smallholder chicken farmers in Tanzania. Tanzania is representative of many countries in Sub-Saharan Africa in that rural production systems comprise the vast majority of total poultry production (estimates from 86–94%) and the historical rate of ND vaccination by households is low [12]. The Tanzania Ministry of Agriculture has estimated that only 22% of households “regularly” vaccinate [13].

### Diffusion of Innovation Theory

In this research, we defined adoption as a gradient consisting of three possible stages, which allows for an understanding of the determinants and barriers for groups of adopters who presumably have unique characteristics. Diffusion of Innovation Theory, developed by sociologist E.M. Rogers, explains how an idea or a product diffuses through a population or social system. Adoption is defined as a series of stages rather than a binary action, and within a population, individuals are in different stages of adoption. Adopters at different stages have different characteristics, and effectively promoting an innovation to a target population requires understanding the characteristics of adopters in their respective stages [14].

### Survey design

The survey was designed to measure the outcome variable, a household’s self-reported stage of adoption, and potential predictor variables, which included household demographics, socioeconomic status, knowledge, attitudes, and practices. The three stages of adoption were awareness of ND vaccines, previous vaccination, and recent vaccination (within four months). Recent use is a proxy for frequency, and a four month time period was used because it is the length of time vaccines are protective against ND on a flock basis [15,16].

The survey was translated into Swahili, and administered as an electronic questionnaire to selected households by pairs of local research assistants, one male and one female. Eligible households had a consenting adult at least 18 years old, currently owned local chickens, or had owned them within the last six months. This research focused on indigenous breed chickens and their crosses, hereafter referred to as local chickens. Two types of ND vaccines are available in Tanzania: La Sota (administered via drinking water, cold chain recommended) and I-2 (administered via eye drops, thermotolerant). Use of either type is considered as adoption in this study. If households reported being unaware of ND vaccines after being asked twice, they were asked an abridged set of questions. When assigning adoption stage, it was assumed households reporting no awareness of the vaccine had never used ND vaccines. All respondents were read a statement explaining the study and possible consequence of participation. Respondents were informed that participation was voluntary. Those choosing to participate provided oral consent in the presence of two research assistants, which was documented within the electronic survey tool. Oral consent was used instead of written consent because it is inclusive to respondents of any literacy level. This research was cleared by the Tanzanian Commission for Science and Technology (COSTECH) through permit No. 2018-32-NA-2015-213. The Washington State University Office of Research Assurances found the project exempt from the need for IRB review (#15068).

A multi-stage sampling approach was used to select households in six villages with the goal of maximizing variation in chicken production practices, access to veterinary services, and household demographics. At the village level, households were randomly selected using a census of heads of households provided by village governments as a sampling frame [17].

### Study sites

**Fig 1:**
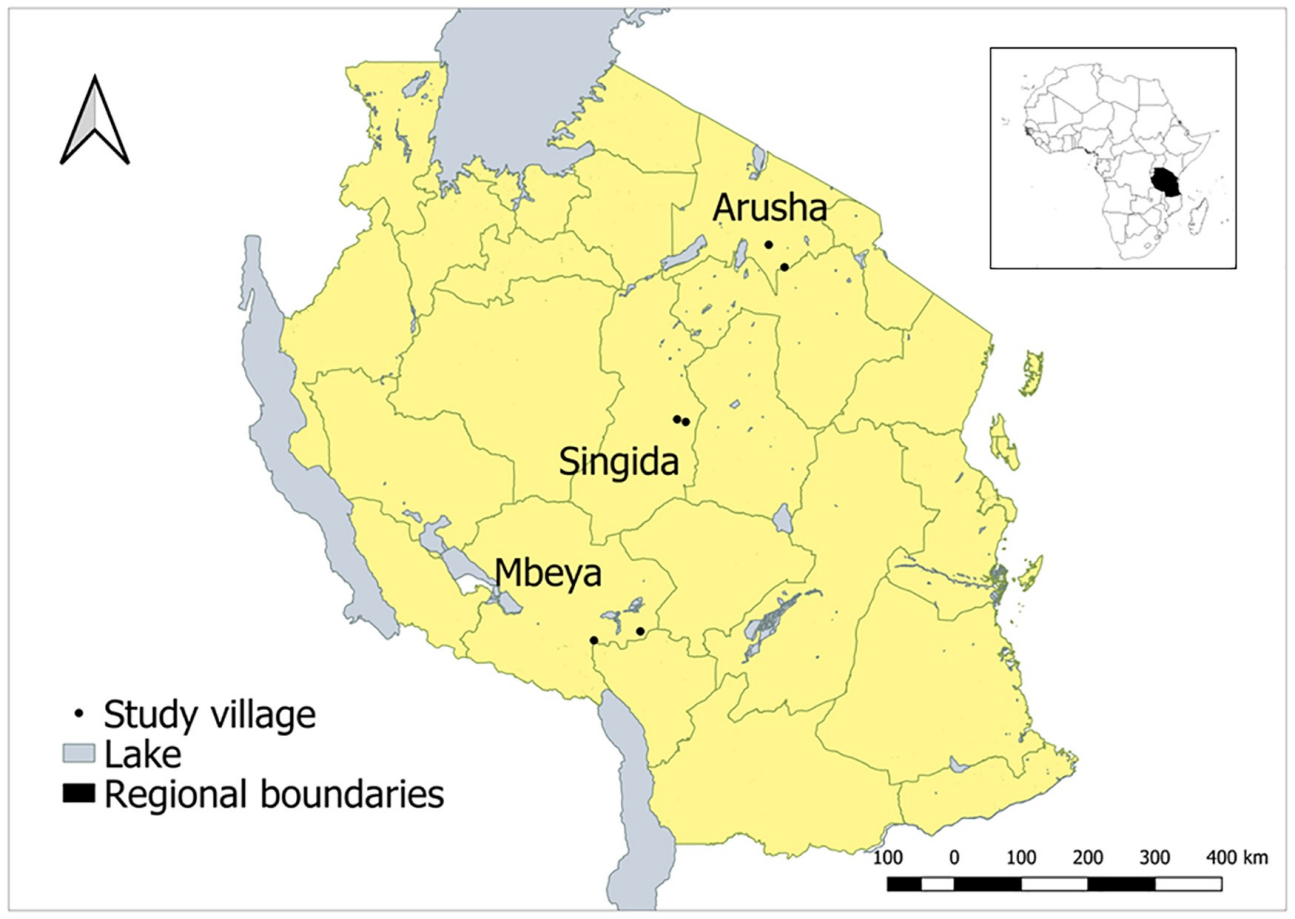
Study site map.

The six villages as shown in Fig 1 span Tanzania’s diversity in geography and climate and have different histories with regards to local chicken production. Arusha Region in northern Tanzania is the home of ethnic groups such as the Maasai and Arusha that have traditionally focused on raising cattle and small ruminants rather than local chickens. Singida Region in central Tanzania has a hot and dry climate, and is known for successful local chicken production in part because of easy access to urban consumers by road and rail. Mbeya Region has a cooler and rainier climate, and local governments have hosted some ND vaccination campaigns in the last ten years. In each region, one peri-urban village (< 25 km from urban center) and one rural village (>25 km from urban center) were chosen for a total of six villages. The villages have between 1,300 and 3,000 residents and varied in their level of access to veterinary services.

### Data analysis

Adoption was modeled as a function of predictor variables using logistic regression. Three models were created using dependent variables representing three stages of adoption. The three stages of adoption were awareness of ND vaccines, previous vaccination, and recent vaccination (within four months), which divides the study population households into four groups. Models 1 and 2 (awareness and previous use) used the full dataset, while model 3 (recent use) was contingent upon previous vaccination which explains the smaller number of observations. Of 535 eligible households, 31 households were dropped due to missing data. Approximately 25 potential predictor variables were considered for each model, and not all variables considered are represented in the best models. The known person predictor is not used in the awareness model because knowing someone who vaccinates indicates awareness of ND vaccine. The full list of predictor variable considered for each model is presented in the S1 File. Model validity was assessed using Akaike information criterion (AIC) and receiver operating characteristic curves (ROC curves) in order to choose a best model. Generous criteria were used to bias selection outcome to retain moderately associated variables (p<0.2). All analyses were conducted using Stata 15 [18].

## Results

We present descriptive statistics about the study population, and the output from three logistic regression models to identify determinants and barriers influencing household decisions to adopt ND vaccines. The data are provided in S3–S5 Files.

### Study population

The study was designed so the enrolled households would be representative of smallholder farmers throughout Tanzania. Bias was minimized by a high consent rate of 99% (759/766). Descriptive data of the study population is presented in Table 1. Farming is the main economic activity for 85% of households, and 45% of households reported off-farm income in the last month. Household wealth in the form of livestock is summarized using Tropical Livestock Units (TLUs). The TLU is a metric developed by the Food and Agriculture Organization (FAO), which allows for the combination of multiple species of livestock into a weighted measure representing total body weight and potentially market value [19]. Households in Arusha own significantly more livestock than households in Mbeya and Singida. Fifty-nine percent of households across all regions own livestock other than chickens. There were no widespread community vaccination initiatives in any of the study villages with the exception of one village in Arusha in which a NGO provided vaccine to a limited number of households enrolled in a chicken-raising group. The majority of households purchased the vaccine themselves (51%, 136/265), followed by 25% who went to a location where the vaccine was brought and vaccinated themselves. Only 14% of households reported receiving the vaccine the last time from a community vaccinator.

**Table 1:** Household and decision-maker characteristics by region, (range and standard deviation for continuous variables).

	Arusha	Mbeya	Singida	All
Flock size *(M)*	13.5 (313, 28)	11.5 (106, 13)	13.4 (77, 12)	12.8 (313, 19)
Farming as main income (%)	80.1	83.2	89.6	84.4
Livestock ownership^+^ (TLU) (*M)*	8.8 (144, 15)	2.6 (91, 8)	2.9 (72, 9)	4.7 (144, 11)
Own other livestock (%)	88.0	46.4	42.8	58.9
Limits to increasing flock (%)	93.0	94.6	94.8	94.1
Poultry info from agro vet^++^ (%)	11.4	9.8	4.0	8.5
Access to hatchery chicks (%)	23.3	9.8	13.2	15.3
Female decision-maker (%)	84.8	65.0	48.3	66.0
Female care-taker (%)	84.8	82.0	85.0	83.9
Decision-maker age (*M)*	44.5 (76, 14)	49.4 (64, 15)	47.6 (81, 15)	47.2 (81, 15)

^+^TLU is Tropical Livestock Units

^++^Agro vet refers to a farmer supply shop which carries agricultural inputs and veterinary supplies.

The average flock size in the study is 13 chickens. Ninety-four percent of households cite factors limiting them from increasing the size of their flock. Only 15% of households report access to hatchery chicks. Eighty-nine percent of households report experiencing a ND outbreak in their flock at some time. Across all regions, the person making decisions about local chickens is a woman in 66% of households, but the gender of the decision-maker varied significantly by region. Across all regions, 84% of chicken caretakers are women.

### Stages of adoption

**Fig 2:**
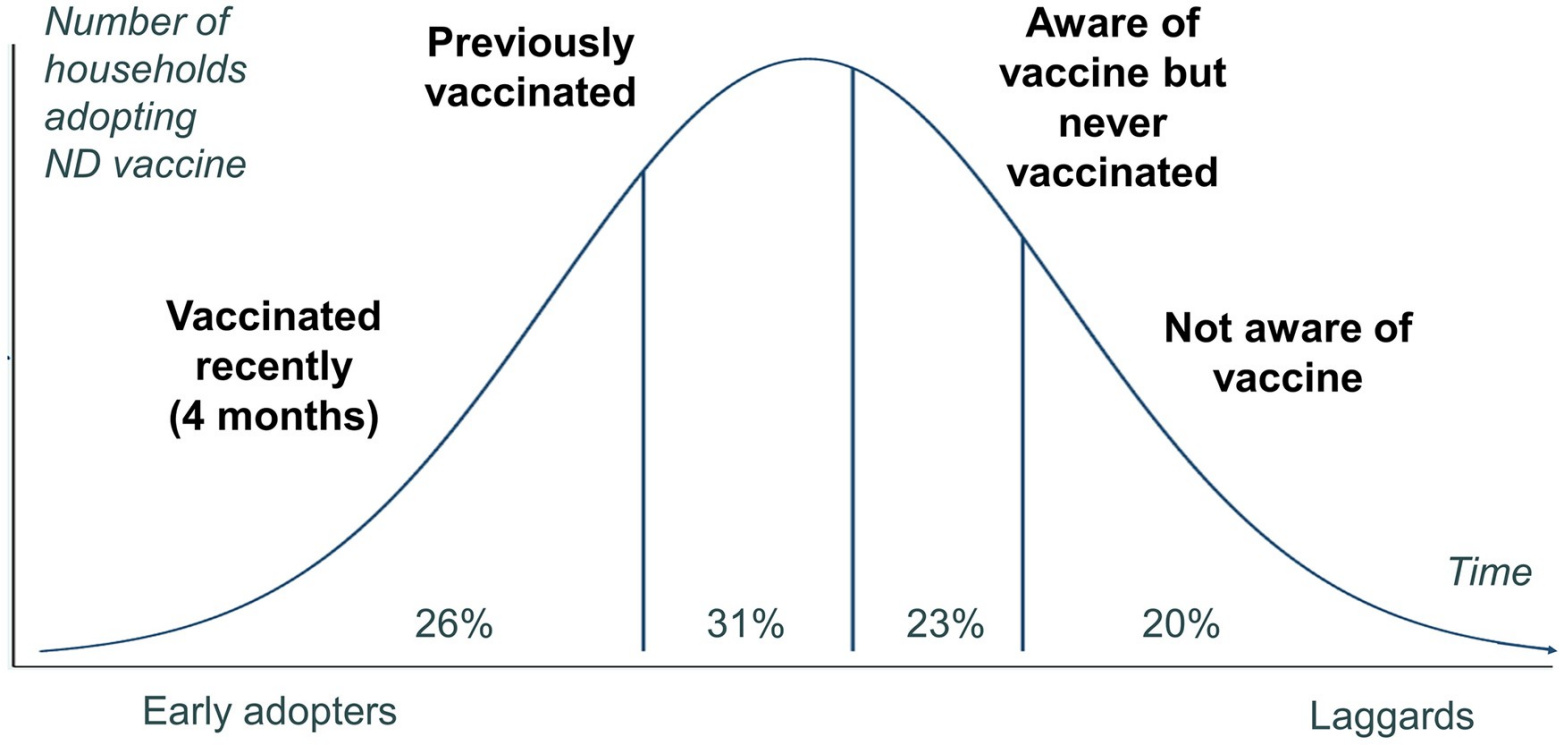
Stages of adoption. Proportions of households in four categories of adoption across all study sites.

The overall profile of the households across all sites with respect to adoption of ND vaccine is shown in Fig 2. While this is a cross-sectional snapshot in time, it can be useful to consider households moving between categories over time as information diffuses through the community. Households reporting recent vaccination are labeled as early adopters of recommended vaccination; they are using ND vaccine consistent with veterinary guidelines while other households in the community lag behind.

Table 2 presents the percentages of households that are aware, previously vaccinated, and vaccinated recently broken down by region. Eighty percent of households report being aware of the vaccine, but only 26% of households report vaccinating within the last four months, a proxy for recommended frequency.

**Table 2:** Summary of three stages of ND vaccine adoption by region, (95% CI).

	Aware of vaccine		Previously vaccinated		Vaccinated within 4 months	
Arusha	69.7	(62.3–76.4)	44.0	(36.5–51.7)	12.0	(7.6–17.8)
Mbeya	81.5	(75.2–86.9)	58.7	(51.3–65.9)	25.5	(19.4–32.5)
Singida	89.0	(83.4–93.3)	67.1	(59.5–74.0)	40.5	(33.1–48.2)
All	80.1%	(76.4–83.4)	56.6%	(52.3–60.8)	25.9%	(22.3–29.9)

### Determinants and barriers

Determinants and barriers and their respective effects on the three stages of adoption are presented in the logistic regression model output in Table 3. A missing value indicates the predictor was not retained in the best model as per selection criteria.

**Table 3:** Determinants of ND vaccine adoption using three logistic regression models, AOR (95% CI).

	(1) Aware of vaccine		(2) Ever vaccinated		(3) Vaccinated recently contingent upon ever vaccinating	
Region			1.39*	(0.99–1.95)	2.22*	(1.53–3.23)
Flock size			1.03**	(1.01–1.05)	1.02*	(1.00–1.04)
Traditional medicine	.55*	(0.30–1.01)	.58**	0.36–0.95		
Know person who vaccinates	N/A		1.32***	(1.14–1.53)	1.37***	(1.12–1.68)
Info community member	2.96***	(1.57–5.57)				
Info seminar	8.32***	(1.80–38.59)	7.72***	(3.01–19.85)		
Belief in vaccine effects	2.35***	(1.25–4.43)	2.12**	(1.15–3.89)		
Knowledge score	1.03***	(1.02–1.04)	1.03***	(1.02–1.03)		
Building materials of home	2.24***	(1.22–4.11)	1.71*	(1.00–2.9)		
Own phone					2.24**	(1.02–4.91)
Land owned			1.21*	(0.97–1.51)	.68**	(0.50–0.91)
Decision-maker education	1.92***	(1.18–3.15)				
Time kept chickens	1.44*	(0.95–2.19)	1.50**	(1.04–2.15)		
Chicken consumption	1.20	(0.91–1.59)			1.42**	(1.03–1.96)
Intercept term	.019	(0.00–0.11)	.01	(0.00–0.03)	.028	(0.01–0.12)
Correct predictions (%)	84.5		76.4		67.1	
Observations	504		504		243	

AOR is adjusted odds ratio, CI is confidence interval, and N/A is not applicable

*** p<0.01,

** p<0.05,

* p<0.1

Region, flock size, and knowing a person who vaccinates for ND are three explanatory variables associated with previously vaccinating or vaccinating recently (Table 3, Models 2 and 3). Singida region consistently had the highest levels of awareness and adoption of ND vaccines, and Arusha region consistently the lowest (Table 2). Regions are coded with Arusha as the reference level, followed by Mbeya, then Singida. Households in Singida region have more than twice the odds of previous vaccination compared to households in Arusha (AOR: 2.58, 95% CI: 1.7–4.0). Households in Mbeya region also have greater odds of previous vaccination than households in Arusha (AOR: 1.83, 95% CI: 1.2–2.8). The pattern continues with households in Singida having over four times the odds of previous vaccination compared to households in Arusha (AOR: 4.38, 95% CI: 2.2–8.8). Region did not have a significant correlation with vaccine awareness in the presence of other variables (Table 3, Model 3).

Flock size, or the number of chickens the household owned at the time of visit, is positively correlated with previously vaccinating and vaccinating recently (Table 3, Models 2 and 3). Flock size was not significantly correlated with awareness of ND vaccine (Table 2). Mean (M) flock size for households unaware of ND vaccines (M: 8.9, 95% CI: 7–11) is not significantly different from the flock size of households that were aware but had not previously vaccinated (M: 9.3, 95% CI: 7–11) as per a two-sided t-test (t (224) = -0.25, p = 0.80).

Households that previously vaccinated have an average of three more chickens (M: 12.6, 95% CI 10–15) than households with awareness of ND vaccines but no experience using the vaccines, which is a significant difference (t (282) = -2.15, p = 0.03). Households that have vaccinated in the last four months as per veterinary best practice have an average of five more chickens (M: 18.8, 95% CI: 14–24) than households reporting previous vaccination but not within four months, a significant difference (t (297) = -2.38, p = 0.02).

**Fig 3:**
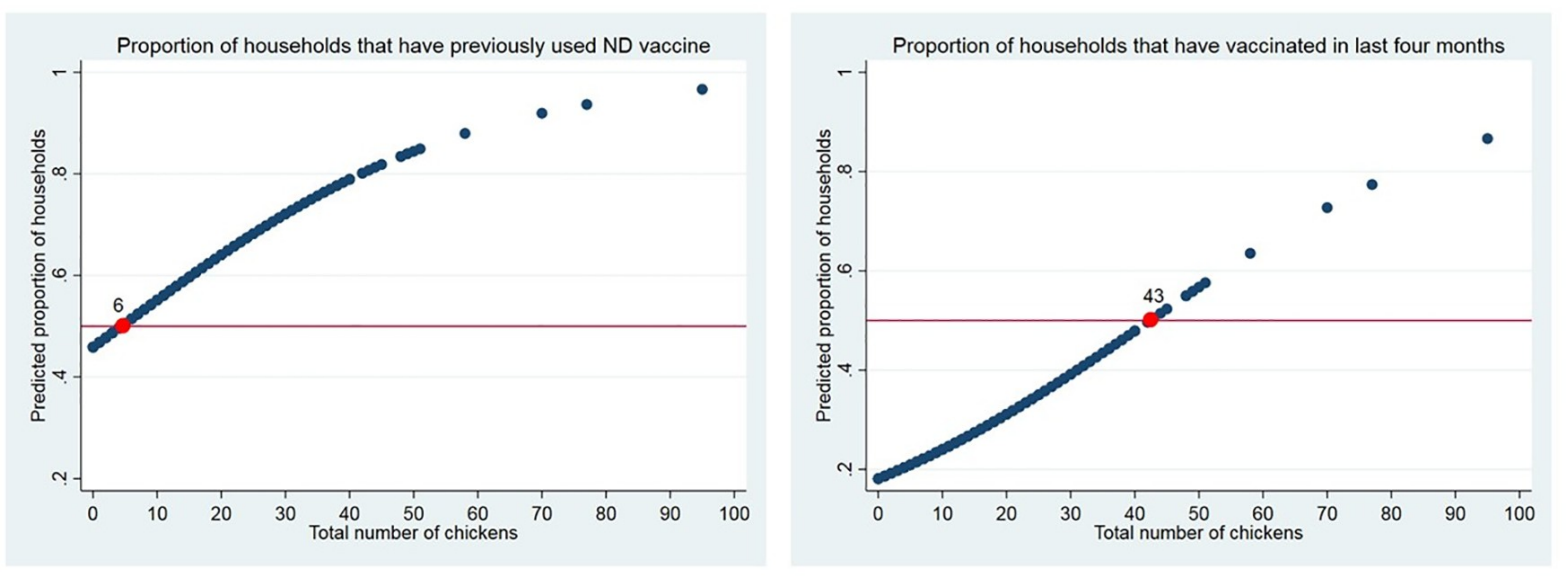
ND vaccine adoption by flock size. Predicted proportions of households that have previously vaccinated versus vaccinated recently (within 4 months) as a function of flock size.

The likelihood of adoption of ND vaccine increases for each additional chicken added to the household’s flock (Table 3). For example, by increasing the flock size by ten chickens, a household is 1.3 times more likely to have previously vaccinated for ND, and 1.2 times more likely to have vaccinated recently. Fig 3 compares the predicted proportions of households that have previously vaccinated versus vaccinated recently as a function of flock size. For a household to have a fifty-fifty chance of previous vaccination, its projected flock size is six chickens. For the same household to have a fifty-fifty chance of *recent* vaccination, the projected flock size increases to forty-three chickens. These numbers can also be interpreted as the projected flock sizes for households if the community is expected to reach 50% of households vaccinating previously or vaccinating recently respectively.

Knowing someone who vaccinates is associated with greater likelihood of previously vaccinating (AOR: 1.32, 95% CI: 1.2–1.5) and recently vaccinating (AOR: 1.37, 95% CI: 1.1–1.7). The known person is most commonly a neighbor (156/307, 50.8%) or a community member (60/307, 20%).

An additional four predictor variables are positively correlated with both awareness of ND vaccine and vaccinating previously (Table 3, Models 1 and 2). These predictors are: receiving information about chickens from a seminar, believing vaccination will lead to a larger flock size, a higher knowledge score based on responses to five true/ false questions about ND and vaccines, and a home built of durable materials such as bricks or cement as opposed to sticks and mud (reference level).

First, households with a member who received information about chickens from a seminar are 8.3 times more likely to be aware of the vaccine (AOR: 8.32, 95% CI: 2–39, p < 0.01) and 7.7 times more likely to have previously vaccinated (AOR: 7.72, 95% CI: 3–20, p < 0.01). In fact, receiving information from a seminar is the strongest adjusted predictor for awareness and vaccinating previously despite only 12% of households reporting seminar attendance. Second, believing someone who vaccinates will later have a larger flock size is associated with greater likelihood of awareness (AOR: 2.35, 95% CI: 1.3–4.4) and of previously vaccinating (AOR: 2.12, 95% CI 1.2–3.9). Third, knowledge score has a smaller but consistently positive effect on likelihood of awareness (AOR: 1.03, 95% CI: 1.02–1.04) and vaccinating previously (AOR: 1.03, 95% CI: 1.02–1.03). Knowledge scores also highlight common misconceptions, for example only 33% of households understood that ND vaccine does not protect against all diseases (177/525). Fourth, households with homes built of more durable materials are more than twice as likely to be aware of ND vaccine (AOR: 2.24, 95% CI: 1.2–4.1) and 1.7 times more likely to have vaccinated previously (AOR: 1.71, 95% CI: 1.0–2.9). Building materials of the home is a proxy for socioeconomic status as wealthier households are able to build with higher quality materials.

Use of traditional medicine is the only variable consistently negatively correlated with likelihood of awareness (AOR: 0.55, 95% CI: 0.3–1.0) and likelihood of vaccinating previously (AOR: 0.58, 95% CI: 0.4–1.0). Forty-five percent of households report using traditional medicine (45.1%, 237/525). The most common traditional medicines are species in the genus *Aloe*; 37% of all households report using *Aloe* species to prevent or minimize symptoms of ND. Households where a woman is the decision-maker for chickens are more likely to use aloe (41% compared to 29% when the decision-maker is a man). Twenty-one percent of households report using both traditional medicine and vaccines to address ND. Land ownership, coded categorically as four levels based on acreage owned, increases the likelihood of a household vaccinating previously (AOR: 1.21, 95% CI: 1.0–1.5) but decreases the likelihood of vaccinating recently (AOR: 0.68, 95% CI: 0.5–0.9).

Gender of the decision-maker is not significantly correlated with any of the three stages of adoption, but there are some noteworthy trends. There is a significant relationship between decision-maker sex and awareness of the vaccine in univariate analysis (χ^2^ (1, *N* = 525) = 12.14, *p* = 0.0005), but this relationship drops out in the presence of other variables. Women are more likely to care for chickens than to have decision-making control over them; in this study 84% of caretakers were women but only 66% of decision-makers were women. Lastly, and perhaps most significantly for interventions, the extent to which women are making decisions about local chickens in the households depends on region. In Arusha Region, 85% of decision-makers are women, compared to 65% in Mbeya Region, and only 48% in Singida Region. In other words, a woman in Arusha is six times more likely to make decisions about the household’s chickens than a woman in Singida (unadjusted *OR*: 5.98, 95% CI: 3.6–10.0).

Not all variables considered are retained in the final three adoption models, either because they exceed a p-value of 0.2 when regressed in a univariate logit model against the outcome variable, or because they exceed a p-value of 0.2 in the presence of other model terms. Absent variables include household income in the previous month, livestock wealth converted into Tropical Livestock Units (TLUs), and the age and gender of the primary person responsible for making decisions about chickens in the household. Three variables unique to model three, recent vaccination, are not retained in the final model: sharing vaccines with another household, availability of vaccines when the household wants them, and payment (whether the household paid or received vaccines for free).

## Discussion

This study examines the behavioral and economic drivers underlying household decisions to adopt or forgo ND vaccination with the goal of improving vaccine coverage and livestock productivity.

Our estimates of flock size and vaccine coverage are similar to those reported by the Tanzania Ministry of Agriculture in the 2007/2008 National Agriculture Census. In our study population, the mean flock size was close to 13, compared to 11 reported by the census. Similarly, our finding that 26% of households had vaccinated within four months is consistent with prior government reporting that 22% of households “regularly” vaccinate [13] and confirm the low rate of vaccine adoption. There may be some seasonal variation in vaccination rates caused by farmers vaccinating before the season in which outbreaks typically occur. The peak outbreak season varied by region and village according to focus groups, which reduces the likelihood of a systematic bias across the study.

### Knowledge and information

Many campaigns or interventions are designed to change behavior by providing information. In this study, possessing specific knowledge about ND and vaccines as measured by a five question knowledge test (see S2 File)), correlates to higher likelihood of vaccine awareness and previous vaccination. A study by Terfa et al. identified the education level of the decision-maker as a driver of ND vaccine adoption among smallholder farmers in Ethiopia, but in our study, education level was only retained in the model of vaccine awareness [20]. In other words, specific knowledge about ND and vaccines was more predictive than level of formal education. If disseminating correct information can contribute to increasing vaccine use, what are the most effective means of communication and who is the target audience?

Knowledge score responses identified pervasive misconceptions which can be addressed with correct information. For example, only 33% of households knew that ND vaccines do not protect against all diseases. Seminars are a popular way to engage smallholder farmers, but their effectiveness varies depending on the household’s stage of adoption. Attending a seminar was correlated with awareness and previous vaccination, but not recent vaccination, suggesting seminars may be effective for introducing ND vaccines but other factors drive frequent use. A lower cost way to reach smallholder households with important information is the radio. Twenty-two percent of households reported getting information about chickens from the radio (95% CI: 19–26), but this was not a predictor of adoption in any of the three models.

The most effective vehicle identified in this study for communicating information that results in vaccine adoption is a community member who vaccinates. While knowing someone who vaccinates has a smaller impact on vaccine awareness and having ever vaccinated than seminar attendance, if increases the likelihood of ever vaccinating, and most importantly, recent vaccination. The most commonly reported contact was a community member followed by a neighbor, which suggests weak ties to a vaccine adopter may be more important to the diffusion of information about vaccines than familial bonds [21]. The importance of social networks as a source of information has been cited in other studies, including a study of East Coast Fever vaccine uptake amongst Kenyan pastoralists where farmers who learned about the vaccine from a fellow farmer were more likely to adopt [22]. Encouraging information dissemination from successful adopters within the community combined with information campaigns might be an effective way to increase adoption.

### Flock size

Increasing flock size is positively correlated with previous and recent vaccination, but the cross-sectional study design does not allow us to determine whether having a larger flock promotes household adoption, or if vaccination itself results in a larger flock through reduced mortality. Other research indicates both forces affect adoption. Vaccination in rural production settings has been shown to reduce mortality and increase flock size of local chickens [10], which supports a causal relationship between vaccination and flock size. Alternatively, having a larger flock may cause households to vaccinate as households are incentivized to protect a more valuable asset [23].

Flock size can be an indicator of a transition from a subsistence mindset to a business-oriented mindset. When a farmer begins keeping chickens as a business enterprise, they will demand new technologies [12]. Within this study, less than a fifth of households had more than twenty chickens, which may highlight the lack of supportive conditions conducive to scaling up. Supportive conditions include market access and development, mechanisms to cushion risk, available information and inputs, and financial and regulatory services [12]. Almost all households in the study report factors limiting increases to flock size, including household finances, thieves, and limited space. Fifteen percent of households reported access to hatchery chicks, an important input for business model production. Lastly, technical support for local chicken production is a major constraint; only 8% of households had ever received information about chickens from an agro veterinary shop. Interventions creating conditions conducive to smallholder households scaling up their local chicken production are likely to lead to smallholder households demanding and adopting ND vaccines.

The presence of barriers to vaccine access unique to households with small flocks is another argument in support of a causal relationship between flock size and vaccination. One example is vaccines sold in large packages with a minimum of 100 doses [12]. In the absence of a community vaccination scheme, sharing is one way that households access vaccine. Sixty percent of households that vaccinate reported sharing the vaccine with another household the last time they vaccinated (181/304). Another barrier to vaccination for households with small flocks is competing interests that make better use of limited time and financial resources. In Arusha region, households may be choosing to focus on investments in other types of livestock over chickens, which we discuss below. In summary, the correlation between flock size and adoption may be explained by causal forces in both directions.

While adoption decision is most tightly linked to potential benefits of vaccination at the household level, there are potential dramatic amplifying effects if vaccination coverage within a community reaches a threshold where inter-household transmission is dramatically reduced or eliminated. We predict that an average household would need 43 chickens for the community to reach 50% vaccination coverage. This prediction is limited by the small number of households with large flocks in the study, but begins to explore the changes needed to move a community towards herd (here community flock) immunity. Fifty percent vaccine coverage is not necessarily the threshold for achieving herd immunity; the calculation of the effective reproductive number required for a more accurate estimate depends on spatial distance and contact rates within the chicken owning households in the communities, which is beyond the scope of the current study [24].

### Traditional medicine

Traditional medicine alone or in conjunction with biomedicine has a well-established role in smallholder local chicken production in Tanzania, and evidence suggests in the case of ND vaccine adoption, traditional medicine has a competitive relationship with vaccination. Use of traditional medicine is associated with a decrease in likelihood of awareness of ND vaccines and of previous vaccination. Usage of traditional medicine is not a predictive factor in the model of recent vaccination, likely because only 34 of the 237 households that had vaccinated recently reported using traditional medicine (14%).

The most commonly used traditional medicine was *Aloe* species. Waihenya et al. found treating chickens with *Aloe secundiflora* reduced mortality and severity of clinical signs, but had no significant effect on antibody levels of chickens inoculated with Newcastle disease [25]. In a study of broiler chickens, *Aloe vera* used in conjunction with ND vaccine resulted in higher antibody titers [26]. The documented benefits of aloe may explain its widespread usage amongst smallholder households. A significant drawback to using aloe is that even if mortality from ND is reduced, treatment does not prevent an infected chicken from acting as a carrier and spreading the disease. Even traditional medicines with pharmacologically-active compounds must be processed and dosed correctly to avoid the risk of being ineffective or even toxic.

In this study, using traditional medicine decreases likelihood of previous and recent vaccination. Despite evidence of a competitive relationship, 21% of households report using both traditional medicine and vaccine to address ND. Medical pluralism, or the practice of traditional medicine alongside biomedicine, has a long history in indigenous populations within low-income countries [27]. Given the history of medical pluralism and the growing evidence that some traditional medicines such as aloe have positive effects, it may be practical to present traditional medicines supported by empirical studies as a viable tool to be used in conjunction with biomedical solutions such as vaccines.

### Gender

Women more commonly make management and investment decisions about chickens than other livestock, and there is evidence from multiple studies showing when women have greater control over household resources, household spending on food and education increases [6,7,28]. This study provides evidence that while vaccination uptake may not be correlated with the gender of the decision-maker; the extent to which women make decisions about local chickens varies by region. Additionally, the percentage of women caring for chickens was higher (84%) than the percentage of women making management decisions about chickens (66%). A classic division of responsibility in most traditional societies allows women access to local chickens but not necessarily full control over the production tools and benefits [29]. A review of livestock production interventions in developing countries by Leroy and Frongillo suggests that the nature of a woman’s responsibilities in livestock production activities varies widely by region, ethnic group, and production system. Leroy and Frongillo find women’s control over income from livestock production activities is limited [30]. In contrast, women often control over the money resulting from the sale of chickens, and gender-sensitive programming can maximize the economic benefits women gain from production of local chickens [31,32]. Interventions to increase ND vaccine coverage may benefit by taking into consideration the local gender roles in caring for chickens versus having decision-making power over them.

### Socioeconomic status

The remaining predictors driving ND vaccine adoption are important but challenging to address with interventions; they are indicators of socioeconomic status, namely the building materials of the home, land ownership, and cell phone ownership. Having a home built with more durable materials such as brick or cement is a predictor of awareness and previously vaccinating. For smallholder households with seasonal incomes, this proved to be a more reliable socioeconomic indicator than last month’s income, which was not retained by any of the adoption models. In the model of vaccination within the last four months, cell phone ownership is the only indicator of socioeconomic status retained in the model.

Land ownership is another indicator of household wealth, but its relationship with adoption is not consistent across models. Land ownership increases the likelihood of previous vaccination but decreases the likelihood of recent vaccination. While owning land increases household wealth, which may positively affect vaccination rates, keeping chickens is an economic activity ideal for households without large amounts of land. Land wealthy households may be focusing on farming or keeping other livestock with higher land requirements such as cattle, goats, and sheep.

Livestock ownership measured in TLUs, another indicator of household wealth, is not retained in any of the adoption models, but the tendency of Arusha households to own more non-chicken livestock such as cattle, goats, and sheep is notable. Considering that Arusha has the lowest vaccination rates of all three regions, the highest ownership of other livestock, and the highest percentage of female decision-makers, one explanation is that owning other livestock is in competition with local chickens for limited household resources. This may be an example of a barrier for households with small flocks; the opportunity cost of vaccinating a few chickens cannot compete with benefits of investing the same time and money into another household endeavor: cattle, sheep, and goats. We hypothesize the wealth in livestock typical in Arusha region is not a household asset easily translated into inputs for local chicken production because women typically control local chickens, but do not have access to resources in the form of larger livestock often controlled by men.

### Strategies for increasing adoption

The importance of the source of information is the most salient outcome of this research. Seminars are an effective first step at creating awareness of ND vaccine and encouraging vaccination, but not sufficient to move households into a state of adoption where they vaccinate frequently. Knowing a neighbor or community member who vaccinates increases the likelihood of previous and recent vaccination. This highlights the power of early and successful adopters in the community to influence others. Equipping early adopters who are respected and well-connected in the community with professional level information about vaccination could be a powerful way to facilitate knowledge diffusion. The positive correlation between adoption and larger flock size suggests interventions creating an environment conducive to scaling up local chicken production could be effective at increasing demand for ND vaccine. Since many households are using traditional medicines, some of which have known benefits, integrating biomedicine with effective traditional medicines may be effective in some communities. Any intervention will be most effective if focused on chicken dependent households with fewer competing interests such as other livestock or large land acreage. Policies aimed at empowering women should consider that while women often control chickens, the extent of their control might vary widely depending on region, ethnicity, and other factors.

Future applied research respective to improving vaccine coverage could focus on the relationship between vaccine adoption and price of the vaccine, the drivers of the decision to use La Sota vaccine versus I-2 vaccine, the pharmacological efficacy of traditional medicines used to treat livestock diseases, and the factors affecting the extent to which women control income or other benefits achieved through livestock production interventions. One limitation of this study was the decreasing power of the models due to shrinking sample size as we moved to higher stages of adoption. A future study with a larger sample size of frequent vaccinators could add to the body of knowledge of the factors influencing those households.

By focusing on the household decision-making process, this interdisciplinary study identifies determinants and barriers of adoption of a vaccine that through control of disease, has potential to improve the health and productivity of livestock, and deliver benefits at the household and community level.

## Supporting information

S1 File List of predictor variables considered in full model. (DOCX)

S2 File Five question knowledge test. (DOCX)

S3 File Dataset. Stata Dataset using Stata 15.
(DTA)

S4 File Dataset. Excel dataset.
(XLS)

S5 File Dataset codebook. Excel codebook.
(XLS)
