# Supplementary material for: Newcastle disease vaccine adoption by smallholder households in Tanzania: Identifying determinants and barriers
Source: PLoS One. 2018 Oct 24;13(10):e0206058. doi: 10.1371/journal.pone.0206058 (PMC6200240; doi:10.1371/journal.pone.0206058)
Supplement: S1 File — (DOCX) [file pone.0206058.s001.docx]

**S1 File: Predictor variables considered in full models**

The following predictor variables were considered for the full models. Variables were retained if they had a p-value of <0.2 in univariate analyses, and a p-value of <0.2 in the presence of other variables. An x indicates the variable was considered for the respective model, N/A indicates the variable was not considered because it was not applicable.

| **Variable** | **Description** | **Aware** | **Previous vaccination** | **Recent vaccination** |
| --- | --- | --- | --- | --- |
| Region |  | X | X | X |
| Flock | Number of chickens owned | X | X | X |
| Known person | Member of household knows someone outside the household who vaccinates chickens for ND | N/A | X | X |
| Land ownership | Acreage owned by household, categorical | X | X | X |
| Income | Household income last month | X | X | x |
| Own phone | Whether any household member owns mobile phone | X | X | X |
| Building materials | Building materials of home | X | X | X |
| Belief | Will someone who vaccinates have a larger flock? | X | X | X |
| Traditional medicine | Whether or not household uses to treat of prevent ND | X | X | X |
| Urban trips | Trips per month by any household member to urban center, categorical | X | X | X |
| Knowledge score | Responses to five question test (Supplemental Materials 1) | X | X | X |
| Time kept chickens | Time kept chickens, categorical | X | X | X |
| Info community | Have you gotten information about chickens from a community member? | X | X | X |
| Info NGO | Have you gotten information about chickens from an NGO? | X | X | X |
| Info agro vet | Have you gotten information about chickens from an agro vet shop? | X | X | X |
| Info seminar | Have you gotten information about chickens from a seminar? | X | X | X |
| Info radio | Have you gotten information about chickens from the radio? | X | X | X |
| Frequency of information from extension officer | Have you received information about chickens from an extension officer? If so, occasionally or often? | X | X | X |
| Decision-maker sex | Sex of the primary decision-maker for chickens | X | X | X |
| Decision-maker education level | Education level of the primary decision-maker for chickens, categorical | X | X | X |
| Decision-maker age | Age in years of the primary decision-maker for chickens | X | X | X |
| Chicken consumption | Frequency of household chicken consumption, categorical | X | X | X |
| Livestock ownership | Total livestock owned by household converted into Tropical Livestock Units | X | X | X |
| Share vaccine | Did the household share vaccine with any other households last time it vaccinated? | N/A | N/A | X |
| Vaccine available | Is the vaccine available when you want it? | N/A | N/A | X |
| Vaccine payment | Last time you vaccinated, did you pay for the vaccine or receive for free? | N/A | N/A | x |
