# Supplementary material for: Newcastle disease vaccine adoption by smallholder households in Tanzania: Identifying determinants and barriers
Source: PLoS One. 2018 Oct 24;13(10):e0206058. doi: 10.1371/journal.pone.0206058 (PMC6200240; doi:10.1371/journal.pone.0206058)
Supplement: S2 File — (DOCX) [file pone.0206058.s002.docx]

| **SECTION K**: **KNOWLEDGE** |
| --- |
| *Answer the following questions.* |
| **K1** A vaccine can help a sick chicken to get better. |
| 1 = Yes  2 = No  999 = Don’t know |
| **K2** The Newcastle vaccine must be given once per year. |
| 1 = Yes  2 = No  999 = Don’t know |
| **K3** Newcastle disease has no treatment, it only has a vaccine. |
| 1 = Yes  2 = No  999 = Don’t know |
| **K4** The Newcastle vaccine protects chickens against all illnesses. |
| 1 = Yes  2 = No  999 = Don’t know |
| **K5** The Newcastle vaccine may not be effective if it isn’t stored properly. |
| 1 = Yes  2 = No  999 = Don’t know |

**S2 File: Five question knowledge test**

Score was calculated by giving one point for each correct response, and no points for an incorrect or “don’t know” response. The correct responses were divided by 5 to give a proportional knowledge score.
